# PW01-005 – Effects of placebo and colchicine on FMF patients

**DOI:** 10.1186/1546-0096-11-S1-A58

**Published:** 2013-11-08

**Authors:** F Özaltın, Y Bilginer, B Gülhan, I Bajin, Ö Erdoğan, S Özen

**Affiliations:** 1 Pediatric Nephrology & Rheumatology, Hacettepe University Faculty of Medicine, Ankara, Turkey; 2 Pediatric Nephrology & Rheumatology, Sami Ulus Children's Hospital, Ankara, Turkey

## Introduction

The diagnosis of Familial Mediterranean fever (FMF) is basically a clinical one although genetic confirmation is of great help to the clinician. A response to colchicine has been suggested as a diagnostic criterion.

## Objectives

The placebo effect of a drug has never been assessed on FMF diagnosis. We aimed to assess this effect in children.

## Methods

Patients who fulfilled the pediatric criteria for the diagnosis of FMF were included in this study. Demographic and clinical features (attack frequency, features of each attack, etc) were recorded. In first part of the study, patients were randomized in two treatment groups (colchicine and placebo) double blind, with a cross-over study design in 3 months duration.

## Results

A total amount of 50 patients (22 girls, 28 boys) were included. The median age of the patients was 8.5 years (2.5-17.5). 78% of the patients suffered from fever attacks suggestive of FMF every 1-4 weeks. The attack interval of the remaining patients was more than one month. At the time of admission, the median values for ESR and CRP were; 24.5 mm/hr (1-100) and 2 mg/dl (0-31), respectively. Half of the patients (n=25) were randomized to colchicine and the other half (n=25) to placebo. At the unblind period the results were assessed: patients treated with colchicine had lower ESR when compared to placebo in the first phase only (p=0.004). CRP, WBC and SAA levels were not statistically different neither in the first phase nor after the cross-over period. However, the number of attacks were significantly less in the colchicine group (median 0 attack) when compared to the placebo group (median 1 attack) (p=0.011).

In the study group, 13 patients were homozygous, 11 patients were compound heterozygous and seven patients were heterozygous for MEFV mutation. The rest of the patients (38%) did not carry any MEFV mutations.

## Conclusion

The number of attacks were less in the colchicine group however, the lack of difference in the laboratory parameters suggest a marked placebo effect.

## Disclosure of interest

None declared

